# Tiktok, a vehicle for Pro-Ana and Pro-Mia content boosted by the COVID-19 pandemic

**DOI:** 10.1192/j.eurpsy.2021.1862

**Published:** 2021-08-13

**Authors:** G. Lladó Jordan, M.D.C. Díaz García, P. Sánchez Esteban, A. Santos Martín, P. Mediavilla Sánchez, M. Jiménez Cubo, M. Miguel Cano, J.A. Gómez Del Barrio, R. Ayesa-Arriola

**Affiliations:** 1 Idival, Valdecilla Biomedical Research Institute, Santander, Spain; 2 Uemc, Miguel de Cervantes European University, Valladolid, Spain

**Keywords:** TikTok, eating disorders, Pro-Ana, Pro-Mia

## Abstract

**Introduction:**

INTRODUCTION TikTok is a social network (SN) that allows users to share short videos about different issues. Since the COVID-19 lockdown, there has been an increase in Pro-Ana and Pro-Mia videos in this specific SN.

**Objectives:**

OBJECTIVES To know the main characteristics about Pro-Ana and Pro-Mia contents among TikTok users.

**Methods:**

METHODS A search was carried out using uncontrolled language with the term “TCA” (ED in English). The study included only Pro-Ana and Pro-Mia resources in Spanish. Resources under the category “recovery” were excluded. A random sample of 16 TikTok was used, since it is enough to estimate, with a confidence of 95% and an accuracy of +/- 20 percentage units, a population percentage that is expected to be around 20%. The studied variables were images, type of resources, “challenges” and misspelled words.

**Results:**

RESULTS In the sample, 68.75% of the profiles were created upon confinement, 56.25% had more than 500 followers and 68.75% had more than 3000 “likes”. 43.75% included more than 30% of ED advocacy content, 18.75% promoted challenges and 37.5% used misspelled words to avoid SN censorship.

**Conclusions:**

CONCLUSIONS There has been a remarkable increase in ED-related content as a result of lockdown. In turn, the increasing number of users who are part of TikTok reveals that this is a SN that can be associated with ED advocacy.

**Disclosure:**

No significant relationships.

